# Designing a synthetic simulator to teach open surgical skills for limb exploration in trauma: a qualitative study exploring the experiences and perspectives of educators and surgical trainees

**DOI:** 10.1186/s12893-021-01417-7

**Published:** 2021-12-15

**Authors:** L. Heskin, C. Simms, O. Traynor, R. Galvin

**Affiliations:** 1 Department of Surgical Affairs, Royal College of Surgeons in Ireland (RCSI), 2nd Floor, 121 St Stephens Green, Dublin, Ireland; 2 Trinity College Dublin, Dublin, Ireland; 3 University of Limerick, Limerick, Ireland

**Keywords:** Surgical simulation, Upper limb trauma, Surgical training, Full task trainer, Feedback

## Abstract

**Background:**

Simulation is an important adjunct to aid in the acquisition of surgical skills of surgical trainees. The simulators used to adequately enable trainees to learn, practice and be assessed in surgical skills need to be of the highest standards. This study investigates the perceived requirements of simulation and simulators used to acquire skills in limb exploratory procedures in trauma.

**Methods:**

Semi-structured interviews were conducted with an international group of 11 surgical educators and 11 surgical trainees who had experience with surgical simulation. The interviews focused on the perceptions of simulation, the integration of simulators within a curriculum and the features of a simulator itself. Interviews were recorded, transcribed and underwent thematic analysis.

**Results:**

Analysis of the perspectives of surgical educators and surgical trainees on simulated training in limb trauma surgery yielded three main themes: (1) Attitudes to simulation. (2) Implementing simulation. (3) Features of an open skills simulator. The majority felt simulation was relevant, intuitive and a good way for procedure warmup and the supplementation of surgical logbooks. They felt simulation could be improved with increased accessibility and variety of simulator options tailored to the learner. Suggested simulator features included greater fidelity, haptic feedback and more complex inbuilt scenarios. On a practical level, there was a desire for cost effectiveness, easy set up and storage. The responses of the educators and the trainees were similar and reflected similar concerns and suggestions for improvement.

**Conclusion:**

There is a clear positive appetite for the incorporation of simulation into limb trauma training. The findings of this will inform the optimal requirements for high quality implementation of simulation into a surgical trauma curriculum and a reference to optimal features desired in simulator or task trainer design.

**Supplementary Information:**

The online version contains supplementary material available at 10.1186/s12893-021-01417-7.

## Introduction

Simulation as an adjunct to teaching surgical skills in the hospital setting is constantly evolving. Its development is expanding in response to decreasing real operative opportunities secondary to reduced time directives and increasing patient expectations [1, 2]. The importance of simulation as an adjunct to in-hospital teaching is being recognised with increasing evidence of its benefits and transferability to the operative setting [3–6]. Despite increasing evidence and advancement in simulation technology offerings, the uptake has been unbalanced and varied [7]. Enquiry into the barriers for implementation of surgical simulation show the importance of cost, time, access and lack of cultural acceptance or tendency to accept the older apprenticeship model [8]. Simulation-based training not only enables the acquisition of surgical skills and their practice but also assessment of the level of proficiency until competency is achieved [9]. However, if simulation-based training is to be incorporated into training curricula, there is a need for high standards in its implementation, its delivery and in the simulators used for surgical skill acquisition.

The aim in simulation is to sufficiently replicate real-life features so the trainees can acquire surgical skills in an environment similar to the operating theatre set-up [10]. The simulation options to teach open surgical skills include synthetic task trainers, virtual reality simulators, animal parts and human cadavers. All have their merits, but synthetic trainers described are often low fidelity, virtual reality simulators are more applicable to endoscopic surgery, animal models have a different anatomy and there are ethical and facility limitations with using human cadavers, as well as post-mortem changes in tissue properties [11–16]. For open skill acquisition, we feel that synthetic models are the most amenable to design improvements, can represent human anatomy, can have appropriate haptic feedback and do not need a wet lab facility for their use. The reproducibility and consistency of synthetic simulators enable repeated practice and uniformity when used to assess proficiency in high stakes exams.

It is estimated that 3.5 million extremity injuries present to US emergency departments annually [19]. Limb injuries occur in 58% of multiple trauma patients and 1% of these have multiple injures to the extremity [20]. Notwithstanding this, simulators for emergency open skill procedures are poorly represented in surgical skills curricula. For trauma procedures either part-task trainers of limb structures, (such as a bone or a tendon) or human tissue or animal labs are necessary for simulation in this area [17, 18]. Management of extremity injuries often involves a multidisciplinary team such as plastic surgeons, orthopaedic surgeons and vascular surgeons. The consequence of mismanagement can threaten the viability of a limb or affect the morbidity of limb function for the patient [21]. Skill acquisition to repair an injured limb is mainly learned in the hospital setting, and these cases are usually on the theatre list after-hours and the types of injuries are unpredictable. In emergency surgery, it is difficult to schedule practice and ensure all trainees get similar exposure in all geographical hospitals.

There are increasing imperatives to include stakeholders such as educators and surgical trainees in the development and implementation of simulation for surgical skills acquisition. Enquiry into the optimal features of the simulator itself has been explored for existing simulators for open skills in other specialities. Studies have reported that computer-based simulators excelled in guided feedback and synthetic simulators benefited from increased haptic feedback and the incorporation of real surgical instrumentation [22]. To our knowledge there are no studies exploring the perspectives of stakeholders in the optimal features for the effective design of simulators to teach emergency procedures in trauma.

We therefore aim to explore the perspectives of both educators and surgical trainees on the optimal features in an effective simulator for limb exploratory surgery. Because simulators for open skills do not function in isolation and depend on external factors, such as how they are incorporated into a curriculum, their facilitation by educators and incentives to practice, we also aim to explore the attitudes to simulation itself and its implementation.

## Methods

### Study design

A qualitative descriptive approach was adopted for this study. This allowed the examination of the perspectives of the participants on this topic and supported inductively identifying themes from participant statements to generate a rich description of their views [24, 25]. This study was conducted and reported according with the Consolidated Criteria for Reporting Qualitative Research (COREQ) [23] (Additional file 1: Table S3).

### Participants and setting

A purposive sampling technique was used in this study and surgical consultant educators and surgical trainees were recruited in order to yield an information-rich study [26]. A stratified sample was sought from participants from all surgical specialities and countries who had experience of surgical simulation training. Inclusion criteria for participation stipulated that all participants were practicing surgeons, had facilitated or were taught surgical skills using simulators on at least three or more occasions. Exclusion criteria included medical students, non-practicing surgeons and surgeons with no or less than three episodes of exposure to simulation. Initially participants were recruited using advertising on social media simulation and surgical sites, presentations at surgical courses and later by snowball purposive sampling. It was important to get an international perspective from educators both working in a clinical practice and facilitating surgical training at simulation centres. Diversity was sought by approaching educators who ranged from heads of simulation centres to surgical course facilitators and trainee participants who ranged in experience from that of a junior to senior surgical trainees. A global representation was also sought and participation came from European countries like Ireland, Scotland, England, Belgium, Denmark, Sweden, Asian countries such as Turkey and Saudi Arabia and the US. Although limb exploration surgery in trauma is generally the remit of plastic, orthopaedic and vascular surgeons in the bigger level 1 centres, it was important to represent other specialities such as general and paediatric surgery as they often have to deal with decision making around this injury in multi-trauma settings.

### Ethical considerations

Ethical approval was obtained for this study from the Royal College of Surgeons in Ireland (RCSI) ethics committee (REF: 001629). All who volunteered to participate received an information leaflet guiding them to the purpose of the study, how it would be conducted, confidentiality regarding their recorded data and anonymity in the reporting of the interviews. All methods were performed in accordance with the relevant guidelines and regulations. All participants were aware that their taking part was fully voluntary and that they could withdraw from the study at any stage. All participants signed an informed consent.

### Study procedure

One-to-one semi-structured interviews were set up using video calling via Zoom or MS teams (Microsoft). Interviews were conducted from August to November of 2020. Video calling was chosen as the audio-visual approach mimicked the interviewer and the participant being in the same room and allowed more accurate interpretation of the conversation by observing nuances in body language. Field notes were taken in conjunction with the recorded conversations, documenting initial impressions and interesting emerging topics. Participants were asked not to state their name or verbalise any identifying information. Video calling also enabled easier scheduling and access to a wider geographical area [27]. A topic guide was used but open questions were preferred. Although the open questions were informed by the aims of the study and a literature search on the topic, the topic guides were confirmed, clarified or slightly altered after a pilot. In the pilot, two consultant educators and two trainees were interviewed by the principal investigator and these conversations were not included in the study. Topics covered in the interview were inquiries into attitudes to simulation in surgical training in general, followed by exploring their views on how it should be used to finally guiding towards their view on the make-up of a surgical simulator, particularly for the exploration of a limb injury (Additional file 1: Table S1).

All interviews were conducted by the principal investigator (LH), a plastic surgeon, educator, PhD student and designer. The interviewer did not have a previous established relationship with any of the participants and had met only two of them previously on a professional basis. The conversations were audio recorded using a diaphone. The average length of the interviews was 35 min (range from 20 to 47 min). All recordings were transcribed and the participants were given a 2-week window with the option to review their transcripts and make corrections. The transcriptions were then anonymised and acronyms are used to identify participants for the purposes of reporting the findings. Interviews were conducted until no significant new data emerged from the conversations and saturation was reached.

### Data analysis

The audio recorded interviews were transcribed verbatim by the principal investigator. Transcripts were checked for consistency with the recordings and accuracy confirmed. Data was analysed in several iterative cycles, identifying emerging themes and ensuring the inductive approach was data driven and not influenced by the researcher’s presumptions or biases. Data were analysed using a reflexive approach to thematic analysis [28, 29]. Themes and subthemes were refined into a coding structure. Two study researchers (LH & RG) analysed the transcripts independently and the emerging themes were discussed and refined as a team. The team were composed of a plastic surgeon, a physiotherapist and a design engineer and this amalgamation of diverse backgrounds ensured clarity in the understanding and interpretation of the data. The principal investigator continually maintained a reflective diary and analytical notes on refinements and revisions. Any disparity in the interpretation of codes were resolved by discussion and final coding was achieved by consensus. The final list of codes reflected both the diversity and patterning in the interpretation of the data. The data were finally checked, reviewing themes against codes and against the original data ensuring they related to each other and similarities within themes were not duplicated.

## Results

A total of 11 educators and 11 surgical trainees consented to be interviewed. The clinical areas of specialty of the participants as well as country of current practice are represented in Table 1.

**Table 1:** Clinical specialties of educators and trainees

Educator speciality	Country	Trainee speciality	Country
General surgery	Ireland	Colorectal	Saudi Arabia
General surgery	Scotland	Colorectal	England
General surgery	Turkey	General surgery	Ireland
General surgery	Northern Ireland	General surgery	Ireland
Colorectal	Belgium	Paediatric surgery	Ireland
Gynaecology	Ireland	Plastic surgery	Ireland
Cardiothoracic	Denmark	Urology	Ireland
Paediatric surgery	Sweden	Orthopaedic surgery	Ireland
Orthopaedics	Ireland	Urology	Ireland
Colorectal	United States	General surgery	Ireland
Plastic surgery	Ireland	Plastic surgery	Ireland

Three major thematic categories emerged from our thematic analysis of the educator and surgical trainee’s commentary.

### Theme 1. Attitudes to simulation

“It makes sense” (Ez).

“Courses can bring surgeons together from different hospitals and countries to share experiences” (Tw).

(E = Educator, T = Trainee, the second letter is a random letter assigned to each of the participants).

There was an overwhelmingly positive response to the use of simulation among all participants, some regarding it as integral, relevant, intuitive and that it should be mandatory. It was considered a great way to start to learn a procedure and it supported deliberate practice. Ethically they felt that it was not really negotiable that a certain amount of the training happens outside the operating theatre.

“Patients do not accept that a surgeon does a procedure on them for the first time” (Ex).

“I think it is unethical to practice on patients, especially when stress levels are high when you are starting your training” (Ew).

Participants felt this safe environment was optimal for learning, practicing new and infrequent cases. It also provided a space for pre-practice or a warmup for more complicated cases or operative planning prior to entering the operating room.

“Simulation good for trouble shooting a problem for a task you would not normally do independently” (Tu).

“Simulation (is) good for pre-practice of skills which allows you to concentrate on higher cognitive skills such as situation awareness” (Es).

There is a lack of balance between the number of surgical trainees and the number of cases with changes in working time directives and a change in patient expectations. Some educators felt that they were performing procedures because their trainees are not experienced enough and had a low number of index procedures in their logbooks. They felt simulation may be able to address this and also have the capacity to train a larger number of trainees on the same procedure over a shorter period of time and increase their confidence.

“Simulation is useful where it is difficult to build up numbers at the workplace” (Tr).

“You can progress on it” (Ts).

“Simulation (is) good for learning difficult versions of the procedure, new procedures, especially untried and tested procedures or modifying techniques” (Es).

Most participants viewed simulation as an important adjunct to clinical learning and an optimal place to practice using surgical instruments, setting up and inserting implants and mastering complex technology such as robotic consoles.

The majority of participants felt simulation made sense, however some acknowledged that the uptake of simulation vary due to some barriers. They highlighted reasons for lack of buy-in or resistance to simulation may be the belief that the Halstead apprenticeship model is better and some may feel that simulation is simply not like the real thing, that the fidelity is low and the expense is high. They also felt that those that trained with simulation themselves tended to be believers.

“Concerns there are limitations to it, feels different to the real thing” (Ev).

“Somethings cannot be simulated such as the nuances of dissecting out surgical planes” (Tw).

Other highlighted barriers were that trainers run busy practices and may be reluctant to give up time in the operating room where they believe the environment is more real and where there is patient contact. Access to simulation and logistics were seen as potential barriers to both educators and trainees. Some trainers may be intimidated by aspects of the technology such as virtual reality simulators.

“Those who did not have simulation as part of their training believe there is no substitute for in vivo operating” (Eq).

“Dynamic, a simulation session may not account for the random un-foreseen variables that come up in real life”. (Ty).

It is felt that non-advocates would need a demonstration of the benefits of simulation, particularly with objective data showing improvement over time, less mistakes, faster performance and fewer complications. However, others felt exposure to simulated learning or observing improvements in their own trainees would be better than promoting it abstractly.

“Hard to prove simulation is better and one reason is that a trial might put patients on the line” (Tr).

“Need public awareness and an understanding of simulation” (Tq).

### Theme 2 Implementing simulation

“Improvements seen will encourage theoretical endorsement and promote enthusiasm” (Ew).

“Ask the trainees, they are the receivers” (Ts).

Participants felt that improvements in implementing simulation would be the creation of frameworks, where it could be incorporated into the curriculum to increase mastery, precision teaching and fluency. They felt that a learning strategy with set goals according to the learner level, cognisant that trainees learn at a different pace would be optimal. It was noted that psychomotor skills are learned over a protracted period and that this should be incorporated into a strategy. They thought that within a curriculum, setting standards, enabling practice and reaching competency is the aim. They felt that allowances for additional content would be necessary to compensate for the lack of opportunities in some hospitals and the expectation of cross cover in different specialities in district hospitals.

“Simulation should be built into the education framework and not just used ad hock”.(Er).

“Practice like in aviation. So you know it so well, it is second nature to you when you get to theatre” (Tv).

Within the simulation sessions themselves, trainee participants felt that it was desirable for the educator to go through the steps of the procedure, physically correct the trainee’s mistakes, demonstrates the correct way and observe them again. They would welcome an ‘exploratory phase’ to make mistakes first and learn from them. They thought starting with practice on simple task trainers to improve psychomotor skills and hand eye co-ordination made sense before moving to a full procedure where the steps were broken down for the learner. Then extending the session with pre-op draping, post-op dressings and finally adding distractions such as bleeps would enrich the experience. Continued repetition with the option to practice at home after a course would aid mastery.

“A consultant can suggest that a trainee practice a specific task on a simulator and then come back to theatre” (Tz).

“Add cognitive flow and a multitude of inputs that you would get in the operating theatre, you can learn to control the environment and learn additional non-technical skills” (Eq).

Participants felt simulation should start at undergraduate level. Accessibility to simulation and a simulation centre was a concern of both the educator and trainee. They felt availability should be during the working week with dedicated protected time for learning and practicing.

“Having a simulator next to the theatre to have your consultant supervise you between cases.” (Ts).

“Dedicated hours to both learn and practice on a simulator” (Tw).

Although some agreed that simulation could be expensive, especially for virtual reality simulators, others felt it was more expensive to teach some skills during precious operating theatre time. There was a demand for more variety in simulators that were externally validated. It was suggested that simulation centres developing simulations should share resources to be more cost effective.

“Share resources, no point in reinventing the wheel” (Ts).

“More affordable with some competitors between simulation companies” (Tx).

They felt the power of progression in learning in a simulated environment relies on specific feedback tailored to the learner.

“Construct alignment, once you have decided the outcomes, intervention starts, then cycle with feedback followed by assessment” (Eq).

“Objective ways to measure more nuanced things like handling tissues, the way they suture, did you identify this structure, preserve another” (Tr).

Meaningful feedback by an experienced facilitator was viewed as necessary for improvement. This was not just for learning the steps of the procedure but for correct use of technology and instrument and tissue handling. There were mixed views on whether a facilitator should be continually standing beside the learner after the task was demonstrated.

“Demonstrate, walk away, reduce stress, do not supress their learning, after an hour come back” (Ew).

“Needs to be a certain amount of check-up and corrections when practicing on a simulator” (Ts).

Not all facilitators were seen as equal or consistent and some seem to be more observant and more inclined to explore what the trainee is thinking while performing a task. It was felt to be important for facilitators to be trained by those who excelled, by teaching the skills of observation and formative feedback.

“Some facilitators are very astute and can detect reasons why trainees are struggling” (Tp).

It was felt that all feedback should be constructive with clear endpoints and after correcting where the learner has gone wrong, the facilitator should revisit the learner after they have had time to practice. The formative feedback given could be guided by a checklist but it was felt that picking up nuances like ergonomics, tissue handling and testing working memory would be important. In addition to this, trainees felt it would be important to encourage, coach and share ‘tips and tricks’ from the facilitators experience.

“An experienced facilitator knows when to interject and encourage” (Ez).

“Objective ways to measure nuanced things like handling tissue, the way I sutured, did I identify a structure, did I avoid another.” (Tq).

Participants reported that the types of objective feedback coming from the simulators themselves should be haptic feedback, visual feedback and barometric feedback. It was observed that most of these objective metrics are incorporated into virtual reality or computer based simulators which are capable of giving printouts of the learner’s hand movements or rate their errors but it was felt that some of these endpoints were not necessarily meaningful for improvement.

“Good to have an instrument that stopped working when used incorrectly” (Tr).

“Don’t think virtual reality simulators are so self-instructive to allow trainees to learn on their own in a safe way at present” (Ew).

Most agreed that it would be challenging to automate feedback within a synthetic simulator for open surgery and there would be a high dependency on a facilitator observing the skills. However, some suggestions included either live interactive video sessions where the facilitator is feeding back remotely or recorded sessions that are later assessed by an expert blinded to who was performing the skill. Other technological advanced ideas such as the use of hand movement monitors, google glass recording and comparison with experts and the incorporation of sensors into surgical instruments were suggested.

“Video live feedback on the simulator, you don’t want them learning the wrong thing” (Tz).

“Cold immersive technology using VR glasses gives a better user experience” (Ew).

It was felt that peer-to-peer feedback would be of benefit and that this feedback should be directed at both the main operator as well as the assistant when many operations are done as a team. It was felt that the main operator’s view and the assistant’s view of the operative field are different for the same operation and that instruction videos should reflect this. One educator felt that learning from multiple videos of the same operations by consultant experts mimicked how sports people learn their skill. Equally revisiting a video of your performance with your facilitator mimicked methods used in sports coaching.

“Pairing up at a simulator, surgery is done as a team” (Ts).

There was consensus among educators and trainees that there is a competitive culture in surgery. However, they did not feel the metrics displayed after using a VR simulator was a strong motivator for simulators use and practice in of itself.

“Despite all the metrics in VR simulators, trainees are not using them more” (Ep).

They thought ways of igniting a more competitive streak would be to see the performance scores of their peers, working up to different levels in the simulator or the incorporation of gamification. Serious gaming modules were seen to be a strong method of internal motivation. Many educators and trainees felt that not being allowed to progress in the operating theatre, unless you had achieved proficiency in the simulation lab would be a particularly strong external motivator. Suggestions to aid this were to create a ‘simulation logbook’ or app or obtaining a certificate when proficiency was achieved.

“Virtual reality, augmented reality and serious gaming are important for new generation integration and engagement” (Ex).

Participants acknowledged that setting standards for accuracy and defined goals and objectives to achieve by a certain time would incentivise simulation practice. They felt real logbook gaps of index procedures that needed to be achieved in their syllabus could be addressed through simulation. Many felt that the timing of the simulated experience must be relevant and relate to opportunities they were getting in the hospital.

“If one had to submit video evidence of their progress” (Tr).

Other motivators included access to simulation and the allotment of protected time to practice. Some simply wanted to be encouraged by their trainers. Some acknowledged that transferability would be difficult to prove but if they saw improvement in themselves, it would encourage them to use it.

“Demonstration that you were getting more fluid and faster would be an incentive” (Tw).

### Theme 3. Features of an open skills simulator

“Capacity to be rebuilt for additional use” (Cw).

In the initial phase of simulator design, participants reported that it was important to identify the purpose of the teaching session and the needs of the trainee group and this could be supplemented by doing a needs analysis. They felt a content expert who knew the critical steps, the danger points and the consequences of any errors in a procedure was critical but also felt that the designer should dissect the anatomy in a lab themselves prior to developing a prototype.

“A clinician who has done the procedure many times must be part of the team” (Cq).

They felt pre-recorded material displaying anatomy and surgical steps were recommended via websites, eLearning, apps, podcasts, review of videos of experts work, should be provided with the simulator. Pre-briefing, setting goals according to the learner’s level and outlining specific outcomes to achieve would seem to enrich the experience. It was felt to be important to have a detailed instructional video with the simulator, any associated technology be an easy plugin and that instruments and consumables, such as grafts, came with the kit.

“Superimpose steps, like the digital simulators do onto physical models, spell out the sequence of steps, ‘colour by numbers’” (Tx).

Participants reported that the physicality of the simulator needs to look and feel real, have fidelity in its anatomy and tissue handling and have good haptic feedback. Participants felt that features including being robust, durable and unbreakable were important. Practice considerations were that the simulator was easy to set up, tear down, clean up and store. They felt it was important to be light and portable for possible home use, yet still have the weight of a real limb.

“Aesthetic reliability makes it more believable” (Cy).

“How it performs is more important than what it looks like or fidelity” (Cp).

Within the limb simulator, it was emphasised that it was important for the injured area to be in context and positioned near normal uninjured muscles, vessels and nerves. They felt this would aid in teaching how to visualise with wound extension and identify injured structures within the limb for their repair. Although clear anatomy was important when exploring the wound, they felt that identifying the structures should not be so obvious. Once the blood and contaminants were cleared it can still be difficult to distinguish structures and they felt this ambiguity would be more real.

“Important that tissues do not look too different to each other, testing the ability to read visual ques” (Cr).

There were practical and more technically challenging suggestions when it came to the individual structures in this simulator. They felt structures should be surrounded by loose connective tissue.

“Ability to dissect surgical planes” (Cz).

As regards bleeding, participants suggested that the simulator should have the capability to have a pulsatile bleed, to clamp the artery, use diathermy, to perform and check an anastomosis. More ambitious suggestions included the ability to remove a clot, use volume of blood loss as a metric, check reperfusion distally in the limb and perform an angiogram. Participants felt the decision making that goes with more complex injuries, such as tissue loss and the application of a vein graft, should be enabled. They thought skin should be represented with edges that were ischemic, with questionable tissue quality. A limb with bone fragments and simulated imaging to match was suggested. If cost was not an issue the participants wish list would be the inclusion of moving fingers to test tendon repairs, twitching muscles and a simulated electrical response when checking intact nerves.

“Related to the in-vivo experience, looks like the procedure, not some abstraction of it” (Tu).

Participants emphasised that the fidelity of the structures and their behaviours while being repaired should be closely aligned with the materials used to make them. They felt that appearance and tactile elements were important but were more concerned with performance. Most had experience with materials such as silicone, plastics and latex used in casting or 3D printing. More advanced suggestions included the use of cultured human skin from research labs or the use of surfactant to make tissues slip over each other realistically. In general, they suggested materials that were elastic, spongy, not too forgiving and had similar tissue turgor and malleability. Ultimately, while manipulating or suturing the simulated materials they wanted them to react like real tissue in terms of having the same pressure, weightiness and not to cut through too easily.

“If the synthetic material does not feel like the real tissue it represents, it may affect your skill negatively in the future” (Ts).

“Materials that respond to a ligature or cautery” (Tv).

Cost was a concern to those interviewed and favoured low running cost and service. Replaceable parts in the form of replacing repaired structures or inserting a removable module within the arm were suggested as solutions. Initially using low cost task trainers for individual structures before going on to use the full task trainer they felt was sensible.

“You want the detail in the anatomical location you are working on” (Tt).

“Cost of replaceable parts more concerning than the initial outlay” (Cr).

“Trainee will never get use out of it if it is too expensive” (Ct).

Participants viewed the full task trainer as being more suitable for more mid-level registrars in plastic, orthopaedic and vascular surgery. Although they felt it would be perfect for use by a team, where the junior might start the procedure and the more senior surgeon take over for more definitive repairs. However, there were some concerns of how relevant it would be for more senior surgeons, for both learning and practice.

“For more experienced trainees, they have experience handling real tissue and may not be interested in a synthetic simulator unless there is more to it” (Ty).

“Depends on level, junior trainees may enjoy working abstractly in a “B &Q” box with abstract tasks but there is a trade-off between cost, fidelity, reproducibility for more senior trainees” (Tr).

The trainees in this study particularly wanted more difficulty in the scenarios as they progressed, they wanted the opportunity to simulate anatomical variants, unpredictable complications and challenging technical scenarios. On a practical level, they felt each simulator should have multiple scenarios within the one simulator or less expensive models that are different to each other with both frequent and infrequently encountered scenarios. They thought that for each skills course the trainee is not just exposed to the same injury but it would be better to go through a different decision making process each time. Despite the acknowledgement that it is a safe environment to make mistakes, some felt there should be consequences for operations going wrong in the simulated setting, and to learn from this and feed it back to subsequent scenarios. This would both increase technical skills and decision-making skills.

“Different scenarios, surprise them, some cases nothing to be repaired” (Cx).

“Need to add the unpredictability you get in real life” (Tp).

They felt that creating scenarios of increasing complexity could be inspired by real patient files, recorded complications or using malpractice reports. Time dependant cases like an ischemic limb would challenge the learner but also to allow decision making to evolve over the procedure.

“Procedure becomes more advanced, throw in curveballs and see the reaction” (Cv).

“If you have a common easy case in the simulator each time, they won’t be able to deal with real life different patient profiles or complications.” (Tu).

“Simulator can be adapted for different tasks” (Cr).

They felt progression would be enhanced by suitable inbuilt feedback. Specific to the limb simulator it was felt that graphical representation of the force while repairing structures would improve skills, a ‘buzz warning’ when you were too close to important structures and time and instrument pathway measurements were really just suitable to the more junior surgeon in training. They felt the more senior surgeons would need to get feedback when mistakes were being made in real time and given direction with ‘tips and tricks’ from experienced facilitators. This could be done remotely with video and reinforced by ‘end of product testing’ like testing an anastomosis.

“A simulator that teaches a basic procedure but that can give a “warning light” where a decision has to be made to a challenge within it” (Cz).

“See the consequences of doing it wrong and progress with this” (Ts).

The responses of the educators and the trainees in this qualitative study were remarkably similar. The only difference was the emphasis by the trainees on filling their surgical logbooks, getting protected time for training, requested more complexity in the scenarios of the simulator. They also communicated much more detail on the type and kind of feedback they would expect in a simulated environment. When detailing required features in a simulator, they tended to have more suggestions to enable safer operating.

“Get a warning buzz when you are close to an important structure or about to make an error” (Tw).

They also suggested some practical additions to a simulator such as adding associated X-ray imaging, adding an inbuilt physiological reaction to bleeding and ergonomic features.

“Correct ergonomic position to mimic theatre in terms of the position of the simulator and the structures inside” (Ts).

## Discussion

This study explored the experiences and perceptions of surgical trainers and trainees regarding the use of simulation generally and the design of a synthetic similar for open limb trauma. The view that the incorporation of simulation is an important adjunct to the acquisition of surgical skills was evident in this study. The benefits of simulation as a safe learning environment were acknowledged and the use of this environment to address shortcoming that both educators (lack of trainee experience) and trainees (practice of infrequent procedures) were experiencing in the hospitals was clear. Our findings correlate with another study exploring the facilitators and barriers to the use of simulation. Similarities between the studies include the positive view of simulation, the belief that simulation should be mandatory and the benefits of simulation in practicing unfamiliar scenarios. Barriers included access and cost, and the limited collaboration with other deaneries to share expertise and facilities [8]. In our study, both educators and trainees saw simulation as having the potential to address the gaps in training where opportunities are just not presenting themselves in the hospitals, at least in adequate numbers. Demonstrating surgical competence in the hospital setting involves the demonstration over time of attaining an increasing number of index procedures under decreasing levels of supervision with the addition of summative assessments such as performance based assessments (PBA) [30]. It seems intuitive that a suggested simulation logbook and proficiency assessments on a simulator by a more independent assessor could supplement this. In the future, there will be a lot more dependency on simulation to test and certify surgeons as they are assessed for skills competency. Further development of more complex, high quality simulators may allow the ‘concept of creating proficiency profiles using simulators’ [31]. An improvement in the type of simulator options, their realism and evidence that assessment in the simulation setting is equal would be desirable.

No matter what simulator you have, most of the conversations in this study explored better ways for its incorporation and use in training. There were several referrals to frameworks, curricula, learning strategies. Some saw the strategy at a surgical training body level and some at a task level. Frameworks have been adapted for teaching surgical tasks and many have similar elements. In a sequential, progressive, modular approach to curricular development and once the appropriate cognitive skills set is acquired, simulator-based training would be used to translate cognition into motor behaviour, and the trainee would practice to proficiency [32].

The cost of running simulation will always be multifactorial. The more expensive resources are the laboratory, the simulation running and service costs and the cost of facilitation by educators. One participant questioned if it was not more expensive to teach skills in the real setting. When teaching necessary operative skills to junior trainees the procedure does take longer but it would also be interesting to investigate if simulation use to skill proficiency reduced errors, expensive insurance claims and potential cost of increased patient morbidity.

The majority of participants wanted simulators of appropriate fidelity and performance to teach the task. There was a clear recognition that this was not to be achieved at any cost in that it would be better to have an affordable simulator. However, the participants were very descriptive in the type of material quality they would expect such as elasticity, turgor and malleability. There were reasonable practical concerns regarding durability, reusability and ease of service storage and setup. Functional fidelity was more important than visual realism but they did think that there needed to be enough visual ques to enable buy-in by more senior trainees. In the literature there is much more written about design considerations for computer based simulators than synthetic based ones for surgical skill acquisition. These studies also refer to the importance of functional fidelity where the tissue reacts and functions more like the real thing [33]. Another study looking at synthetic simulators used in neonatal surgery listed the characteristics the authors would look for in simulators. Important considerations that emerge from this study included fidelity in three domains, accuracy of anatomy, tissue texture and in the replication of procedural difficulty [34].

In our study there was an overwhelming need, particularly from trainees, to have more complex, challenging scenarios that incorporated real patient complications to prepare them for real-life cases. There are specialities such as neurosurgery were small deviations in complex scenarios can cause serious consequences [35]. Some simulation companies have designed anatomical variants such as the position of the appendix (Limbs and Things^@^), however it is uncommon.

In regards to suggested features in a limb simulator, concerns were expressed regarding the lack of certain features in existing simulators such as the basics of having instruments supplied or more complex features as having consequences to complications. Although ascertaining the learning outcomes from getting a procedure task analysis from an expert seems obvious prior to a simulator design, many other authors feel that doing a Cognitive Task Analysis (CTA) is more superior [33]. In reference to the exploratory limb simulator, most participants put an emphasis on the ability to control and repair blood vessels and the realism that goes with an operative field obscured by blood in trauma. This is an aspect of all simulation types at present, unless it is live animal. There are studies showing methods of pumping simulated blood in a human or animal cadaver [36]. Most synthetic simulation offerings who have simulated blood vessels under the skin are prohibitively expensive.

Participants recognised that having an entire limb with the injured structures surrounded by uninjured anatomy was an important design consideration. This approach enhances the learning experience when compared to learning the skills on basic part-task simulators. However, some participants also felt that such a simulator might be more suitable for middle grade trainees, highlighting the importance of mapping the complexity level of technique to the skill level of the trainee.

The strength of this study is that it sought an international view of both the educators and the trainees themselves on the use of simulation and the features of a simulator. We recruited trainers with a broad range of surgical experience. However, we acknowledge that there is limited representations from trainers and trainee participants from specialities more closely aligned to open limb trauma including plastics, orthopaedics and vascular surgery. The nine trainees recruited from Ireland came from different parts of the country but they may have had a similar experience of simulation to each other. Our advertising prior to recruitment did encourage participation from surgeons both for and against simulation, to get a balanced view. If any participants were for simulation we asked then in the interview what they thought the barriers were for non-advocates. In view that the majority of participants were in favour of the use of simulation, we acknowledge that some of the barriers reported are inferred from conversations with non-advocates.

The results of this study give an interesting perspective not only on the views of the participants on simulation in surgical skill acquisition and its use but also on the details requirements they would hope for in the design of a limb exploratory simulator. Much of the requirements would be desirable in any surgical simulator (Additional file 1: Table S2). The results raise further questions regarding how to achieve some of the requirements of the simulator at a reasonable cost. Having multiple versions of the same simulator or incorporation of different procedures in the same simulator will inevitably make a more complex design. Although the majority of the responses from both educators and participants agreed that an increased fidelity and probable increased cost for the more senior trainees would be desirable, it would be a challenge for current material and design processes to achieve this. The details in the suggestions of the physical characteristics and the incorporation of feedback and motivational features will inform future simulator designers. Future research in the importance of the various suggestions in regards to improved educational effectiveness to achieve learning outcomes and enabling the trainees to reach proficiency will be needed.

## Conclusion

Creating an extremity simulator for the acquisition of skills to repair an injured limb involves a team approach. Ultimately, the surgical trainees and educators are key stakeholders and this study serves to explore their views in relation to simulation and its incorporation into the practical surgical skills training setting, as well as the features they would hope for in a limb exploratory simulator. The results of this study will positively inform important considerations in its design. Trainees and experts have similar views on all aspects of simulation in this study.

## Supplementary Information

**Additional file 1: Table S1.** Question topics. **Table S2.** Features of a simulator, a summary. **Table S3.** COREQ (COnsolidated criteria for REporting Qualitative research) Checklist.

## Data Availability

The datasets used and/or analysed during the current study available from the corresponding author on reasonable request.
