# Supplementary material for: Designing a synthetic simulator to teach open surgical skills for limb exploration in trauma: a qualitative study exploring the experiences and perspectives of educators and surgical trainees
Source: BMC Surg. 2021 Dec 15;21:417. doi: 10.1186/s12893-021-01417-7 (PMC8672577; doi:10.1186/s12893-021-01417-7)
Supplement: Supplementary file 1 — Additional file 1: Table S1. Question topics. Table S2. Features of a simulator, a summary. Table S3. COREQ (COnsolidated criteria for REporting Qualitative research) Checklist. [file 12893_2021_1417_MOESM1_ESM.docx]

**Additional file**

Table S1. Question topics:

| Questions | prompts |
| --- | --- |
| Views on simulation | Why good/bad |
|  | Estimate facilitators/barriers |
| Views on the implementation of simulation for surgical skill acquisition | Teaching methods |
|  | feedback |
|  | Practice in simulation centre/home/ incentives |
| Views on what a simulator should incorporate | Effective features |
|  | Look like/performance |
|  | Particular to limb exploration |

Table S2: Features of a simulator, a summary.

| Features of a simulator |
| --- |
| - Durable - Robust, unbreakable. - Flexible - Reusable - Cost effective to buy and run - Replaceable parts - Appropriate fidelity for the learner, realistic - Materials react like real tissues and feel like cutting into tissues - Size, easy to store - Easy to set up, tear down, clean and maintain - Portability, easy to plug into a PC - Reproducible, can be made in high volumes - Can take home to practice - Haptic feedback - Can give feedback, LMS in digital simulators - Have defined goals and standards attached - Associated pre-learning material, anatomy, technique, 3D features of skill - Tuition while using the simulator - Includes instruments, sutures - Ergonomics, can change the angle or raise the height up and down - Increasing difficulty, complications, malpractice scenarios - Related to the in-vivo experience - Can do many different tasks on the one simulator - Can be adapted for different tasks - Can be used by different levels of trainees - Multiple variations of the same procedure - New or infrequent procedure, simulate complications - Increasing complexity - Bank of pathologies in the simulator - Can be adapted to when you have an assistant and without - Interchange among users, multidisciplinary - Can be up-dated, future proofed - All steps of procedure from skin incision to closure - Can prep operating field, insert aesthetic - Can give feedback, metrics printout, graphical score - Auditory or visual feedback when you are close to an important structure or about to make an error - Physical measurement of outcomes i.e. volume of blood - Possible to take a video, visualisation of skill being performed - Can bleed and diathermy can be applied - Externally validated |
| Features of a simulator to teach limb exploration after trauma |
| - Look and feel real - Clear structures and anatomically correct - Contaminated wound with dirt, demonstrate inflammation, necrosis. - Some ambiguity regarding structures (not made artificially colour coded), look real - Bleed , difficult to see, realistic blood volume to emphasise emergency, pumping mechanism for blood, can clamp the artery, ability to test anastomosis, check reperfusion - Include images of bone fragments of vascular scans - Include commonest limb trauma - Include challenges like crushed vessels that may need a vein graft - Ergonomics change the limb position   In a superior model:   - Moving fingers tic hack a tendon repair - Pulse to check blood flow - Muscles twitch - Nerve can be checked with an electrode to check if intact - Connect structures with loose connective tissue |

Table S3. COREQ (COnsolidated criteria for REporting Qualitative research) Checklist

| No. | **Guide questions/description** | **Reported in:** |
| --- | --- | --- |
| 1  2  3  4  5  6  7  8  9  10  11  12  13  14  15  16  17  18  19  20  21  22  23  24  25  26  27  28  29  30  31  32 | **Domain 1: Research team and reflexivity**  **Personal characteristics:**  Interviewer/facilitator: Which author/s conducted the interview or focus group?  Credentials: What were the researcher’s credentials? E.g. PhD, MD  Occupation: What was their occupation at the time of the study?  Gender: Was the researcher male or female?  Experience and training: What experience or training did the researcher have?  **Relationship with participants:**  Relationship established: Was a relationship established prior to study commencement?  Participant knowledge of the interviewer: What did the participants know about the researcher? e.g. personal goals, reasons for doing the research.  Interviewer characteristics: What characteristics were reported about the interviewer/facilitator? e.g. Bias, assumptions, reasons and interests in the research topic.  **Domain 2: Study design.**  **Theoretical framework:**  Methodological orientation and Theory: What methodological orientation was stated to underpin the study? e.g. grounded theory, discourse analysis, ethnography, phenomenology, content analysis.  **Participant selection**  Sampling: How were participants selected? e.g. purposive, convenience, consecutive, snowball.  Method of approach: How were participants approached? e.g. face-to-face, telephone, mail, email.  Sample size: How many participants were in the study?  Non-participation: How many people refused to participate or dropped out? Reasons?  **Setting**  Setting of data collection: Where was the data collected? e.g. home, clinic, workplace.  Presence of nonparticipants: Was anyone else present besides the participants and researchers?  Description of sample: What are the important characteristics of the sample? e.g. demographic data, date.  **Data collection**:  Interview guide: Were questions, prompts, guides provided by the authors? Was it pilot tested?  Repeat interviews: Were repeat inter views carried out? If yes, how many?  Audio/visual recording: Did the research use audio or visual recording to collect the data?  Field notes: Were field notes made during and/or after the interview or focus group?  Duration: What was the duration of the inter views or focus group?  Data saturation: Was data saturation discussed?  Transcripts returned: Were transcripts returned to participants for comment and/or Topic Item No. Guide Questions/Description Reported on Page No. correction?  **Domain 3: analysis and findings.**  **Data analysis**:  Number of data coders: How many data coders coded the data? Description of the coding tree: Did authors provide a description of the coding tree?  Derivation of themes: Were themes identified in advance or derived from the data?  Software: What software, if applicable, was used to manage the data? Participant checking: Did participants provide feedback on the findings? **Reporting**:  Quotations presented: Were participant quotations presented to illustrate the themes/findings? Was each quotation identified? e.g. participant number.  Data and findings consistent: Was there consistency between the data presented and the findings?  Clarity of major themes: Were major themes clearly presented in the findings?  Clarity of minor themes: Is there a description of diverse cases or discussion of minor themes? | Methods.  Methods  Methods.  Female  Methods.  Methods.  Methods.  Introduction.  Methods.  Methods.  Methods.  Methods.  None.  Methods.  Methods.  Results.  Methods.  No.  Methods.  Methods.  Methods.  Methods.  Methods.  Methods.  Results.  Methods.  From the data.  None.  Not done.  Results.  Results.  Results.  Results.  Within major theme reports. |

(Developed from: Tong A, Sainsbury P, Craig J. Consolidated criteria for reporting qualitative research (COREQ): a 32-item checklist for interviews and focus groups. International Journal for Quality in Health Care. 2007. Volume 19, Number 6: pp. 349 – 357)
